# Use of Feedback Data to Reduce Surgical Site Infections and Optimize Antibiotic Use in Surgery: A Systematic Scoping Review

**DOI:** 10.1097/SLA.0000000000004909

**Published:** 2021-04-20

**Authors:** Shalini Ahuja, Nathan Peiffer-Smadja, Kimberly Peven, Michelle White, Andrew J. M. Leather, Sanjeev Singh, Marc Mendelson, Alison Holmes, Gabriel Birgand, Nick Sevdalis

**Affiliations:** ∗ Center for Implementation Science, Health Service and Population Research Department, King's College London, UK; † Faculty of Medicine, Department of Infectious Disease, Imperial College London, UK; ‡ Florence Nightingale Faculty of Nursing, Midwifery & Palliative Care, King's College London, UK; § King's Center for Global Health and Health Partnerships, School of Population Health and Environmental Sciences, King's College London, London, UK; ¶ Department of Anesthesia, Great Ormond Street Hospital, London, UK; || AMRITA Hospital, Kerala, India; ∗∗ Division of Infectious Diseases & HIV Medicine at Groote Schuur Hospital, University of Cape Town (UCT), Cape Town, South Africa.

**Keywords:** antibiotics, audit and feedback, implementation science, patient safety, surgical site infection, surveillance

## Abstract

Surgical site infection (SSI) prevention remains significant, particularly in the era of antimicrobial resistance. Feedback on practices and outcomes is known to be key to reduce SSI rates and optimize antibiotic usage. However, the optimal method, format and frequency of feedback for surgical teams remains unclear. The objective of the study is to understand how data from surveillance and audit are fed back in routine surgical practice.

**Methods:**

A systematic scoping review was conducted, using well-established implementation science frameworks to code the data. Two electronic health-oriented databases (MEDLINE, EMBASE) were searched to September 2019. We included studies that assessed the use of feedback as a strategy either in the prevention and management of SSI and/or in the use of antibiotics perioperatively.

**Results:**

We identified 21 studies: 17 focused on SSI rates and outcomes and 10 studies described antimicrobial stewardship for SSI (with some overlap in focus). Several interventions were reported, mostly multimodal with feedback as a component. Feedback was often provided in written format (62%), either individualized (38%) or in group (48%). Only 25% of the studies reported that feedback cascaded down to the frontline perioperative staff. In 65% of the studies, 1 to 5 implementation strategies were used while only 5% of the studies reported to have utilized more than 15 implementation strategies. Among studies reporting antibiotic usage in surgery, most (71%) discussed compliance with surgical antibiotic prophylaxis.

**Conclusions:**

Our findings highlight the need to provide feedback to all levels of perioperative care providers involved in patient care. Future research in this area should report implementation parameters in more detail.

Postoperative infections and antimicrobial resistance (AMR) are indivisible global threats.^1^ Global guidelines have been issued to mitigate the risk of postoperative infections,^2^ including surgical antibiotic prophylaxis. The global incidence of surgical site infections (SSIs) is estimated by WHO to be 3% to 50%, depending on the type of surgery. In Europe, SSIs represent 17% of all healthcare associated infections (HAIs).^3^ Other postoperative infections that occur beyond the incision site (eg, pneumonia, urinary tract infection, sepsis) also represent a substantial burden and safety concern in perioperative care.^4^ In low and middle income countries SSIs account for about 60% of total HAIs.^3^ Despite preventive measures, postoperative infections are a major cause of morbidity in Africa with postoperative complications occurring in 18.2% of 10,885 patients in a prospective observational cohort study.^4^ Consequently, up to 60% of surgical patients receive antibiotics postoperatively whilst in hospital and up to 50% are discharged with a course of antibiotics. Many of these prescriptions may be inappropriate,^5,6^ thereby feeding the AMR problem in a vicious cycle.^7^ AMR resulting from antimicrobial overuse threatens society's long-term capacity to effectively deliver high-quality perioperative care and healthcare overall.^8^

One commonly used “family” of interventions that is applied within perioperative care to improve antibiotic selection and timing is surveillance methods. Surveillance methods effectively rely on regular collection of data around a specific intervention (eg, timing and selection of antibiotics to be used perioperatively). Within hospitals, surveillance methods are a key component of infection prevention and control and have been shown to drive down infection rates.^9^ However, the mechanism that drives such decreases of postoperative infection rates remains poorly understood. Surveillance alone, that is, carrying out audits of SSIs and their possible causations, does not lead per se to automatic improvement in infection rates.^10^ What has been shown in the literature is that for surveillance to improve outcomes it needs to be paired with effective feedback mechanisms to frontline providers, so they can change their behavior in light of the quantitative data presented to them.^11^ Epidemiologic surveillance coupled with feedback to providers is known in the evidence base as “audit-and-feedback.” Specifically in relation to the use of antibiotics, use of “audit-and-feedback” has been found effective in changing how antibiotics are prescribed perioperatively; improving patient outcomes; and reducing health care costs.^12^

There is however a glaring gap in the evidence base. The details/specifics of *what information; in what format;* and *with what frequency* that surgeons and other physicians involved in perioperative care should be provided with is not clearly defined. Without this evidence, it is not possible to effectively change practice and optimize perioperative antibiotic use to improve surgical outcomes at scale.^13^ Whilst this is not addressed, “audit-and-feedback” interventions will remain sub-optimally applied and of variable effectiveness in surgery.

Implementation science methods can potentially help reduce this evidence gap. Implementation science is a recently established interdisciplinary field, which aims to develop methods and techniques that accelerate the implementation of evidence-based practices within healthcare. Implementation strategies are defined as methods or techniques used to facilitate the uptake of evidenced interventions or programs into clinical practice.^14^ Implementation outcomes are akin to clinical outcomes^15^; they are geared towards assessing the efficacy of an implementation process or strategy and they can be assessed quantitatively, via objective measures and validated scales. Implementation science approaches have started to emerge in the surgical literature – see for example, a recent implementation review of the global implementation processes of the WHO surgical safety checklist in this journal.^16^ A similar assessment of implementation strategies and implementation outcomes could help optimize implementation of “audit-and-feedback” for SSI prevention and antibiotic optimization in surgery – but it has never been carried out to our knowledge to-date. Therefore, this study tries to address this gap by understanding the efficacy of the implementation process in the reported studies.

We report a systematic scoping review of the evidence for how “audit-and-feedback” interventions to reduce SSIs and optimize antibiotic usage in surgery are implemented, and the efficacy of the reported implementation strategies.

## METHODS

The review protocol was registered in the Open Science Framework database and can be accessed at https://osf.io/8nq.

The review was carried out in accordance with standard scoping review methodology.^17^ Scoping review methodology was appropriate in light of the lack of detailed specification of the intervention of interest – that is, “audit-and-feedback” in the context of perioperative antibiotics use. In summary, the recommended stages of a systematic scoping review include: (1) defining the research question, (2) identifying relevant studies, (3) selecting relevant studies, (4) charting the data and collating, and (5) summarizing the results. The application of these stages to this review is summarized below.

### Research Question and Review Scope

The research question is “how are ‘audit-and-feedback’ interventions applied in surgery?” Specifically, how data from surveillance and clinical audits target antibiotic usage in surgery, SSI preventive measures, SSI rates, are reported and utilized in routine perioperative practice The recommended SPIDER (Sample, Phenomenon of Interest, Design, Evaluation, Research type) format was used to structure the research question.^18^ The study sample (hospitalized patients receiving surgical care), phenomenon of interest (feedback from SSI/antibiotics surveillance and/or audit interventions or programs), design (any reported study design), evaluation (any reported evaluation) and research type (any published literature) were included in the search.

### Search Strategy

A systematic search for original articles in the MEDLINE and EMBASE databases was performed. The search was conducted between June 2019 and September 2019. The selected timeline of evidence was from 1974 till September 2019. Search within the databases was limited to keyword and Mesh word search. Search terms were devised and tailored to each database (for details, see Supplementary material- file 2), covering 5 search fields: (1) surgery, (2) SSIs, (3) antimicrobial use, (4) surveillance/audit, and (5) feedback. In addition, the reference lists for all selected full-text articles and related reviews were scanned to derive any further relevant articles. Backward and forward citation and “incognito” Google Scholar (Google LLC, Mountain View, CA) reviews helped to further identify relevant articles.

### Study Selection

Inclusion and exclusion criteria were developed through an iterative process, as recommended for scoping reviews.^17^ Screening was performed at 2 stages: title abstract screening stage and full text screening stage.

During the title and abstract screening stage, any published studies targeting surgical in-patients and focusing on clinical outcome feedback (ie, SSI rates) or clinical process measures (ie, audits of SSI preventive and antimicrobial stewardship interventions) and feedback from antimicrobial stewardship programs/interventions were initially documented. All articles in the English language were screened.

Inclusion and exclusion criteria were tested for reviewer agreement by 2 primary study reviewers (SA: implementation scientist; NPS: infectious disease specialist) who jointly applied them to the first 10% of articles identified by the search at the title and abstract screening stage of the review. The criteria were found viable due to the consistency in the agreement between the reviewers on how to apply inclusion and exclusion criteria. The primary reviewers shared the screening of all studies of this review. Any subsequent disagreement between the reviewers were discussed and resolved by consensus with input from a third reviewer (GB: senior infection prevention and control specialist).

### Study Quality Appraisal

A quality appraisal was performed using the Integrated Quality Criteria for Systematic Review of Multiple Study Designs (ICROMS) which unifies, integrates, and refines quality criteria for quantitative and qualitative study designs.^19^ The tool provides scores to studies and has mandatory criteria and minimum scores for each study type. The ICROMS allocates a “yes” (2 points), a “no” (zero points) and an “unclear” (1 point) score to each one of the ICROMS criteria. The minimal score requirement was based on the study design as presented in the Supplementary file -1 Table A.^20–22^ As per ICROMS, the minimal score requirement for inclusion of studies in the review was as follows: RCTs = 22; controlled before and after studies = 18; uncontrolled before and after studies = 22; qualitative studies = 16; surveys =16; and other designs = 16. None of the identified studies were excluded because of low quality based on these criteria.

### Data Extraction and Analysis

Included articles were subsequently exported to the Endnote software (Clarivate Analytics, Philadelphia, PA) to remove duplicates. Later, the articles were exported to Rayyan software^23^ where the title and abstract screening was performed by the 3 reviewers.

During the full text screening stage data were extracted using a spreadsheet to record “characteristics of the included studies,” “feedback methods,” “implementation strategies”^14^ (See Supplementary material- file 2), and “implementation outcomes”^15^ (see supplementary material -file 2). The full text data extraction form was tested on 10 randomly selected articles before full application to all studies. Data extraction was performed independently by the first 2 researchers and the extracted sheets were then compared by the third researcher.

We applied a number of well-established implementation science frameworks to the retrieved studies. Firstly, we mapped the implementation strategies reported in the reviewed studies onto the evidence-based Expert Recommendations for Implementing Change (ERIC) implementation strategies taxonomy.^24^ The ERIC group applied systematic evidence review and expert consensus methods to develop a taxonomy of 73 distinct implementation strategies used in healthcare. These are further grouped into nine domains: use of evaluative strategies, provision of interactive assistance, adaptation and tailoring to the context, developing relationships amongst stakeholders, training and educating stakeholders, supporting clinicians, engaging patients and service users, utilizing financial strategies, and changing the infrastructure. We used the ERIC taxonomy of strategies to code the implementation strategies reported in “audit-and-feedback” interventions targeting SSI reduction and optimization of antibiotics in surgical care.

Secondly, we summarized the feedback and other ERIC implementation strategies by dividing the studies into five categories: feedback only, feedback and additional one/ two/ three/ four or more ERIC implementation strategy domains. The clinical and implementation outcomes reported across studies were combined into effective, partially effective and ineffective. A study was coded as “effective” if more than half of all the outcomes improve significantly; “partially effective” if approximately half of the outcomes improved significantly; and “ineffective” when fewer than half of the outcomes improved significantly or when the statistical significance was not reported.

Thirdly, we evaluated the efficacy of the reported implementation strategies, by evaluating the reporting of implementation outcomes. The current “gold standard” framework for implementation outcomes used in healthcare includes the following 8 outcomes: acceptability, adoption, appropriateness, feasibility, fidelity, cost, penetration, and sustainability of an evidenced intervention.^15^ We applied this framework to the retrieved studies, alongside any clinical outcomes these studies reported.

The findings from the included studies were synthesized using a narrative approach, with elements of thematic analysis^25^ and thematic synthesis.^26^ Implementation strategies and outcomes were mapped formally using the implementation science validated frameworks described above.

## RESULTS

### Study Selection

**FIGURE 1:**
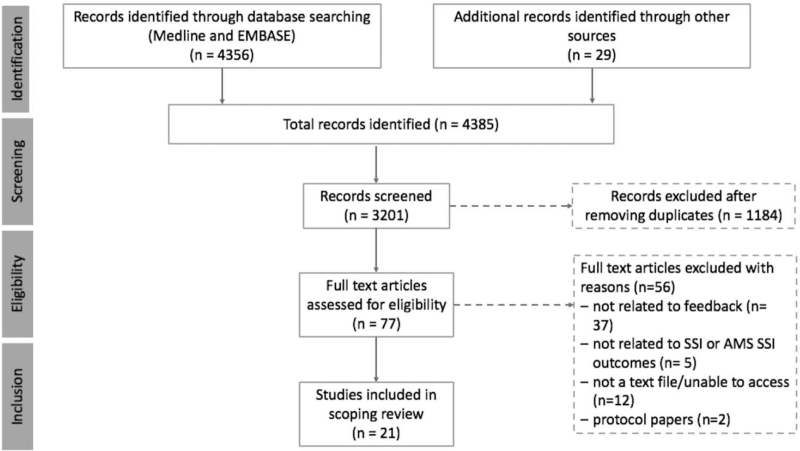
Preferred Reporting Items for Systematic Reviews and Meta-Analyses (PRISMA) diagram.

Database searches in Medline and EMBASE yielded 4356 articles, while an additional 29 articles were identified through other sources, including Google Scholar and expert consultations. Once the duplicates were removed, 3201 records underwent title and abstract screening, which resulted in 77 papers. During the full text screening, 21 out of 77 were identified as focused on feedback strategies and were included in the review (Fig. 1). Out of these 21 studies, 17 focused on SSIs and 10 studies focused on antibiotic use in surgery (with 8 overlapping studies).

### Study Characteristics (Supplementary File -1 Table A)

The majority (86%, 18/21) of included studies were from high-income countries. Forty-three percent (9/21) of publications originated in Europe, 38% (8/21) in Canada and the United States, and 4.7% (1/21) in Australia. Studies done in Nigeria, South Africa, and Egypt were the only 3 studies from low- and middle-income countries. All of the included studies were published between 2005 and 2019, with over 60% published from 2015 onwards. Approximately half of the included studies described single-center interventions, mostly conducted in a tertiary care hospital. The remaining half of the studies were multicenter studies, ranging from 2 to 37 centers, with a median of 3 centers. Sixty-six percent (14/21) of the studies reported were uncontrolled before–after studies, 4 were retrospective cohort studies, 1 was a qualitative study, 1 a mixed methods study, and 1 a systematic review. The systematic review was excluded from the denominator while carrying out various objective analyses, which brings the denominator in objective analyses to 20. However, the systematic review was included in the narrative synthesis as per standard practice in scoping reviews.

All the major surgical specialties were represented in the selected studies, including orthopedic and trauma surgery (n = 9, 45%),^27–35^ general surgery (n = 10, 50%),^28,30,32–39^ gynecology and obstetrics (n = 8, 40%),^31–35,40,41^ vascular (n = 3, 15%),^28,31,35^ urologic (n = 2, 10%),^28,35^ cardiac and thoracic (n = 3, 15%),^28,35,42^ neurosurgery (n = 2, 10%),^28,43^ oncologic surgery (n = 2, 10%),^27,37^ maxillofacial surgery (n = 1, 5%),^35^ plastic surgery (n = 1, 5%),^35^ and pediatric surgery (n = 2, 10%).^23,44^

### Quality of Included Studies (Supplementary File -1 Table B)

Of the 20 intervention studies, 14 used uncontrolled before and after designs and had a median global ICROMS quality score of 17 (IQR: 13–24). The 3 retrospective surveillance studies had a ICROMS score of 11, 20, and 11, respectively. The global ICROMS score for the 3 remaining studies was 2 (1 prospective cohort study^43^), 19 (1 qualitative study^35^), and 26 (1 mixed methods study^42^), respectively.

### Feedback Delivery Mechanisms (Supplementary File -1 Table B)

The included studies provided a variety of surveillance/audit and feedback approaches. Most of the reported interventions (14/21) were multimodal interventions with feedback offered alongside several other implementation strategies also aimed at improving surgical outcomes/processes of care. Mapping the findings onto the ERIC framework revealed that these additional implementation strategies included iterative and evaluative strategies, providing interactive assistance, adapting interventions to the local context, developing relationships among stakeholders, training and educating stakeholders, supporting clinicians, and changing the infrastructure.

Feedback was received in written format in 13 of the 20 studies (65%), either via email, posters, or meeting minutes. Feedback was presented orally in 3 out of 20 studies (15%). In 10 out of 20 studies (50%), group feedback was received and 8 studies (40%) reported adopting an individualized form of feedback (ie, to individual surgeons or other clinicians). Feedback on various outcomes such as SSI rates, compliance with guidelines, and the indication of antibiotics was provided in the 20 included studies. Fewer (6/20; 30%) studies reported both the recipients and the feedback providers. In most studies (12/20; 60%), feedback was given to the head of surgery departments or separately to the surgeons and anesthesiologists or other prescribers. In only 6 out of the 20 studies (30%), the feedback cascaded down to other frontline staff including operating room staff, pharmacists and nurses. In terms of frequency of reporting, the feedback (with some overlap) was provided on the next day of a clinical case (2/20; 10%), weekly (2/20; 10%), monthly (3/20; 15%), 4 monthly (4/20; 20%), 3-monthly (3/20; 15%), 6-monthly (2/20; 10%) or annually (1/20; 5%). In 4 studies, the frequency of feedback was either unclear or not-reported. Only 2 studies stated explicitly that the clinicians received the feedback more than once.

### Intervention Characteristics Reported Across Studies (Supplementary File -1 Table A)

Among the included studies, the most commonly reported interventions were studies focusing on reducing SSI rates using surveillance methods (45%, 9/20), followed by audits on surgical antibiotic prophylaxis (SAP) (40%, 8/20). Prevention measures included hair removal techniques (15%, 3/20), normothermia (10%, 2/20), skin preparation (5%, 1/20), use of the World Health Organization Surgical Safety Checklist (5%, 1/20), antithrombotic (5%, 1/20), and fluid and electrolyte management (5%) were also reported. Of all the studies included, most (75%, 15/20) but not all provided direct data on SSI rates. Out of those 15 studies, 73% (11/15) reported a significant reduction in SSI rates with the applied intervention based on statistical *P*-values and/or confidence intervals, 13% (2/15) reported no statistically significant change, and in 13% (2/15) of studies statistical significance could not be traced.

Of the 10 studies reporting on antibiotic usage in surgery, 9 mentioned SAP interventions, with most reporting data on compliance with SAP recommendations (77.9%, 7/9). In addition to SAP measures, the daily defined dose, antibiotic cost per procedure and the proportion of new colonization or calculation of the percentage of patients having methicillin-resistant *Staphylococcus aureus* infection were also reported.

### Analysis of Implementation Strategies (Fig. 2 and Table 1)

**FIGURE 2:**
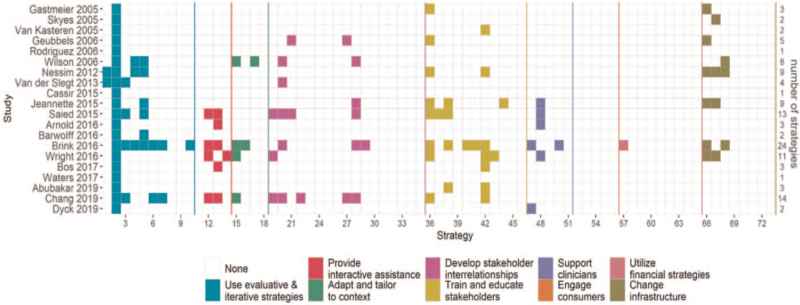
Implementation strategies in the included studies.

Figure 2 describes the ERIC-defined strategies of the 20 included studies (excluding the single systematic review). The median number of strategies reported was three (range 1–24). Approximately 65% (13/20) of the studies reported involving 1 to 5 strategies, 50% (6/12) reported involving 6 to 15 strategies, and 5% (1/20) reported involving more than 15 strategies. Over 50% (37/73) of the 73 ERIC strategies were not reported in any study.

The 2 most commonly reported strategies in the reviewed studies (all of which focus on audit and feedback approaches to reduce infection rates and optimize antibiotic consumption across the surgical pathway) were stakeholder training and education strategies (55%, [11/20] and developing relationship amongst stakeholders 40% [8/20]). These were strategies applied over and above the overarching audit and feedback approach, which was present in all included studies due to the nature of the review. Strategy domains *“providing interactive assistance”* and “*supporting clinicians”* were only reported in six studies. The least commonly used strategies were “*engaging consumers,”* “*utilizing financial strategies,” “adapting and tailoring to the context*.”

The summary of feedback and other ERIC implementation strategies and their effectiveness are provided in Table 1. The correlation between the number of implementation strategies and study effectiveness could not be established due to low numbers hence lack of statistical power. Most of the studies (80%, 16/20) reported improvement in the combined clinical and implementation outcomes.

**TABLE 1:** Feedback and Other Implementation Strategies and Their Effectiveness

	SSI Rates and ATB Use Outcomes		
	Effective^∗^	Partly effective^†^	Ineffective^‡^
Feedback (n = 4)	3	0	1
Feedback + 1 ERIC Implementation strategy domain (n = 4)	4	0	0
Feedback + 2 ERIC Implementation strategy domains (n = 4)	3	1	0
Feedback + 3 ERIC Implementation strategy domains (n = 4)	3	1	0
Feedback + 4 or more ERIC Implementation strategy domains (n = 4)	3	0	1

∗ Effective, if more than half of all outcomes improved significantly.

† Partly effective, if approximately half of the outcomes improved significantly.

‡ Ineffective, if fewer than half of all outcomes improved significantly.

### Analysis of Implementation Outcomes (Fig. 3)

Data on implementation outcomes were extracted and mapped onto Proctor's implementation outcomes taxonomy (which includes appropriateness, adoption, acceptability, feasibility, fidelity, implementation cost, coverage, and sustainability; see Supplementary material- file 2). A minority of included studies (40%, 8/20) reported on implementation outcomes. Except for a study reporting on implementation costs, 87.5% (n = 7/8) of the studies reporting at least 1 implementation outcome reported on fidelity of intervention application (ie, the intervention applied as intended and specified). The elements of fidelity that were reported were almost exclusively adherence and compliance with guidelines.

**FIGURE 3:**
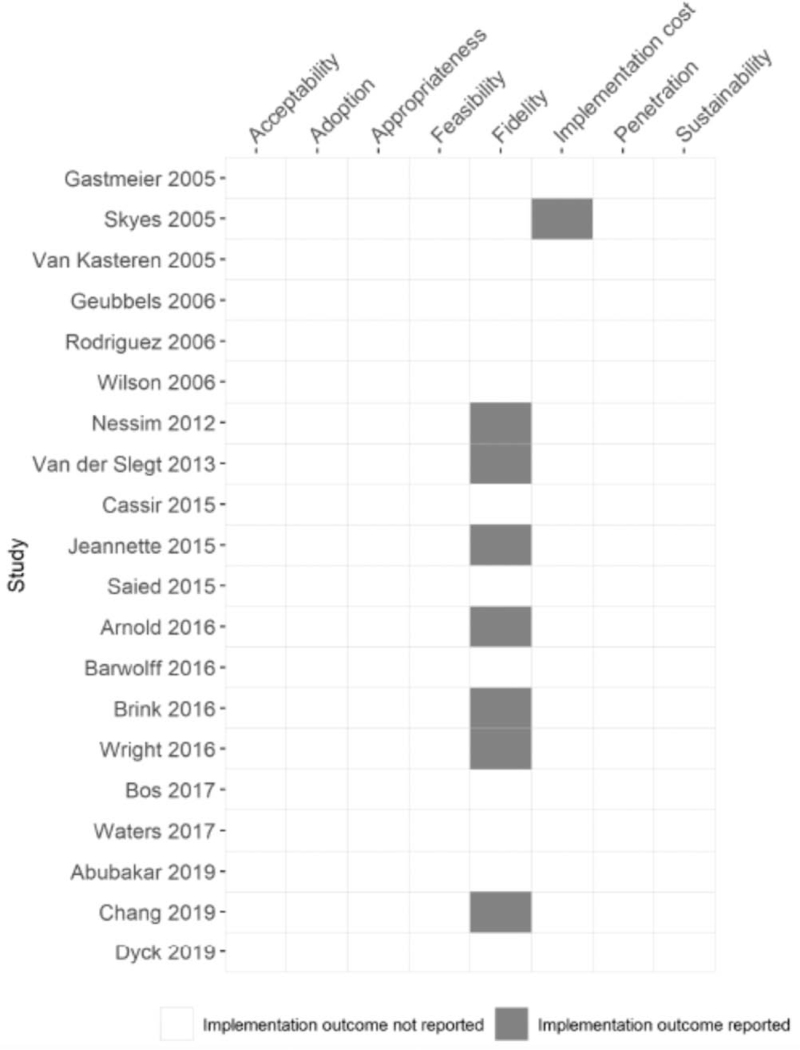
Implementation outcomes assessed in the included studies.

## DISCUSSION

The current study shows that feedback, when delivered, is a part of a multimodal intervention or as a component of a multistrategy bundle. The feedback was received mainly in written format over emails, once every few months, collectively or communicated individually to the head of the departments. Few studies reported that feedback was cascaded down to frontline staff, including operating room staff and nurses. The most frequently reported ERIC implementation strategies were training and education, and developing relationships. These findings are, however, to be interpreted in the context of the included studies, which had a defined focus on audit and surveillance data and their feedback plan aiming to improve SSI rates and ATB use in surgery – rather than as isolated strategies.

The SSI rates, compliance with SAP guidelines, and the indication for using antibiotics were the main outcomes provided in the feedback content. However other strong recommendations by the WHO including surgical hand preparation, surgical site skin preparation and intraoperative oxygenation, were not reported in the feedback content of the studies. The interventions significantly improved the SSI rates in most studies. However, as most of the studies reported intervention “bundles” (of which data feedback was 1 element), the attributable contribution of the feedback process to the improvement in clinical outcomes is difficult to estimate. Various other factors may also play a role in SSI rates, including patient characteristics (eg, absence of comorbidities, nutritional status and smoking), operation factors (eg, surgical wound class including amount of contamination, surgeon's skill, length of operation, use of drains and prostheses), organizational factors (eg, operating room airflow, cleaning and sterilization of equipment, nurse to patient ratio's, and crowding of patients), microbial factors Skin/GI/nasal carriage of micro-organisms; Bacterial virulence factors + drug resistance; and length of preoperative admission that were not taken into account in most studies. Even if it is not statistically a direct cause of clinical outcome improvement, this review and wider evidence suggest that data feedback is a key element which needs to be addressed systematically in improvement strategies.

The feedback process overall yielded a reduction in SSI rates and an increase in compliance with other infection-prevention and infection-control practices. The fidelity was the main reported implementation outcome, mostly as adherence and compliance with guidelines and protocols.

Feedback strategies to improve antibiotic prescription practices and decrease the rate of infection at surgical sites are not well-researched. The current study therefore provides a deeper understanding of how to implement evidence-based interventions, to address higher infection rates and inappropriate antibiotic usage among surgical patients and inspire researchers and implementers to think differently about audit and surveillance strategies in the future. The overall quality and rigor of the study designs, most of which were uncontrolled before–after studies, were found to be low to moderate. The evidence-base remains overwhelmingly from high-income countries, which may be indicative of the gap in research on this subject in low and middle income countries.

Surveillance of postoperative infections and the auditing of practices around infection prevention and antibiotic usage in surgery facilitate the opportunity to drive changes in patient outcomes and reduce health care costs.^9^ In the United States, conducting SSI surveillance through a program marked by the introduction of a feedback system and the involvement of control experts and epidemiologists led to a dramatic 32% decrease in nosocomial infection rates.^45^ However, surveillance in itself does not lead to a reduction in infection rates.^10^ In the last decade, health workers on infection prevention and control and antimicrobial stewardship teams have spent a lot of time pursuing the collection and analysis of surveillance and audit data,^46^ while less effort has gone into utilizing the outputs from surveillance systems to reduce infection rates. To be effective, data should be clinically relevant, and the feedback should be both individualized and aggregated, provided in a timely manner (varied frequency reported in the studies), potentially visually, and available to everyone. Furthermore, it should be delivered in a manner promoting goal-setting and positive deviance principles facilitating quality improvement. Since we could not carry out the statistical analyses on the association of feedback approaches and clinical outcomes, our recommendations on feedback frequency and the feedback delivery process are based on narrative synthesis of the studies involved and the domains of application of the surveillance and feedback strategy as they emerged across studies. Overall, the analysis of the current literature shows the feedback methods to improve infection prevention and antibiotic usage across the surgical pathway are poorly reported. We propose that surgeons can undertake substantial leadership in this area – by leading on specifying what is to be collected and fed back and subsequently leading on improvement efforts, such that surveillance does not become a meaningless “box-ticking” exercise and the data are actually useful to perioperative teams. Substantial literature confirms the importance of surgical leadership for improved care^47–49^; successful implementation of surveillance and actionable feedback programs is an area where such leadership can be demonstrated in practice.

A varied representation of certain specialties—mainly orthopedic and trauma surgery, gynecology and obstetrics, and general surgery—were observed among the included studies. Large bowel surgeries and cesarean sections were the 2 highest contributors of total SSIs per annum in a recent SSI surveillance study.^9^ With regards to the rational antibiotic use in surgery, the current study suggests that approximately 90% of the studies reporting on antibiotic use are SAP-focused, implying that fewer programs may exist that address other aspects of antibiotic therapy across the surgical pathway. Recent studies have indicated that surgical patients are more likely than medical patients to receive antibiotics during their stay in the ward,^8^ thereby contributing to the burden of AMR. It can be argued, therefore, that future research into the use of antibiotics in surgery should not be limited to surgical prophylaxis. Across settings, audits of antimicrobial use, with inherently diverse parameters, have shown improvements.^40^ Changing antibiotic choices are found to be simpler compared to observing improvements in duration and timing of prophylaxis, the latter often being the targets of evolving standards.^34^ Studies have shown that various strategies used to enhance compliance with the timing and the duration of surgical prophylaxis include audit and feedback and educational meetings.^40^ Specifically, feedback, as a strategy was stated to have positive effects on antibiotic indication, decision to shift from IV to oral route and cost savings.^36^

Guidelines for SSI prevention and control measures have been widely developed and disseminated, however the poor adherence to such guidelines is well documented. Amongst other factors, inconsistent reporting of implementation strategies further complicates the issue.^45^ This review, to the best of our knowledge, is the first to retrospectively report implementation strategies using the validated ERIC implementation framework, in the field of postoperative infection prevention and inappropriate antibiotic use in surgery. This review of the ERIC implementation strategies suggested that most studies report the need for training and stakeholder engagement. We found that approximately 50% of the 73 discrete ERIC implementation strategies have not been applied in audit and feedback interventions for SSIs in the reviewed evidence (or at least they have not been reported). These unused strategies, which cover more nuanced approaches in the areas of engaging consumers, providing incentives, and contextualizing the strategies appropriately within individual hospitals or hospital systems, remain to be further explored.

Successful implementation is established by a number of implementation outcomes, including but not limited to fidelity.^50^ Fidelity was the most commonly reported implementation measure in the evidence base, mostly focused on adherence to guidelines. Future research studies might consider the measurement of other fidelity measures and, moreover, other implementation outcomes such as acceptability and feasibility (which are hypothesized in the implementation evidence base to predict adoption) and also medium to longer term sustainability of implementation.

The review has several limitations. Studies assessing the antibiotic use, hand hygiene and other infection prevention control measures at the hospital-wide level were not included. Only two databases were relied upon for the identification of potentially eligible studies, which may not represent the entire body of literature in the field but should still encompass most of the relevant studies. The study did not establish any association between the number of implementation strategies and clinical outcomes because the overall small sample (hence lack of statistical power) and heterogeneous nature of the studies did not enable us to conduct a meta-regression analysis. Many articles may have underreported their implementation strategies, potentially because some articles may have considered strategies to be intuitive and therefore may not have explicitly stated them – hence the review may have been impacted by an unknown reporting bias. Since the statistical analysis on the association of feedback approaches and clinical outcomes could not be carried out, our recommendations on frequency and the process to be followed is based on findings from the narrative synthesis.

## CONCLUSIONS

This literature review highlights a potential gap in the reporting of feedback process while documenting research on SSI prevention and control and antibiotic use in surgery. Future studies on audit and surveillance of infection prevention and antibiotic use in surgery should pay attention to the recipients of feedback, the process employed, and the impact of feedback strategies.

## Supplementary Material

Supplemental Digital Content

## Supplementary Material

Supplemental Digital Content
