# Supplementary material for: Use of Feedback Data to Reduce Surgical Site Infections and Optimize Antibiotic Use in Surgery: A Systematic Scoping Review
Source: Ann Surg. 2021 Apr 20;275(2):e345–52. doi: 10.1097/SLA.0000000000004909 (PMC8746888; doi:10.1097/SLA.0000000000004909)
Supplement: Supplemental Digital Content [file ansu-275-e345-s001.docx]

**Supplementary file -2**

**Appendix 1: Search strategy for electronic databases (Medline and EMBASE)**

| Search strategy for electronic databases (Medline and EMBASE) | |
| --- | --- |
| **Surgical Site Infections** | Surgical site infection OR surgical infection OR post-operative wound infection OR wound infection OR prevention and control OR post-operative infection OR infection control |
| **Antimicrobial Stewardship** | Antibiotic prophylaxis OR (prophylactic AND antibiotic) OR antibiotic stewardship OR antimicrobial stewardship OR antibiotic prescribing |
| **Surveillance** | Surveillance OR Infection Surveillance OR Audit and Surveillance OR Audit and Feedback OR Operating Standards OR Medical Audit OR Clinical Audit OR Follow-up |
| Surgery | Exp. Operating theatre OR Exp. Operating rooms  OR Intraoperative procedures OR intraoperative period OR Preoperative period OR preoperative procedures OR Perioperative period OR perioperative procedure OR Postoperative period OR postoperative procedure Exp. Surgery (which includes - exp decompression surgery/ or exp spine surgery/ or exp nephron sparing surgery/ or exp laparoscopic surgery/ or exp pancreas surgery/ or exp cardiovascular surgery/ or exp spinal cord surgery/ or exp cancer surgery/ or exp endovascular surgery/ or exp gastrointestinal surgery/ or exp minimally invasive cardiac surgery/ or exp cornea surgery/ or exp off pump surgery/ or exp geriatric surgery/ or exp cerebrovascular surgery/ or exp natural orifice transluminal endoscopic surgery/ or exp vein surgery/ or exp uterine tube surgery/ or exp cytoreductive surgery/ or exp ear nose throat surgery/ or exp biliary tract surgery/ or exp major surgery/ or exp nose surgery/ or exp pediatric surgery/ or exp intestine surgery/ or exp coronary artery surgery/ or exp thymus surgery/ or exp refractive surgery/ or exp glaucoma surgery/ or exp bypass surgery/ or exp throat surgery/ or exp meniscal surgery/ or exp hand surgery/ or exp laser refractive surgery/ or exp orthopedic surgery/ or exp liver surgery/ or exp minor surgery/ or exp heart surgery/ or exp kidney surgery/ or exp thyroid surgery/ or exp stapes surgery/ or exp cardiac surgery intensive care unit/ or exp aneurysm surgery/ or exp periprosthetic surgery/ or exp microvascular surgery/ or exp urologic surgery/ or exp thoracic aortic surgery/ or exp breast surgery/ or exp reconstructive surgery/ or exp knee surgery/ or exp urethra surgery/ or exp face surgery/ or exp trachea surgery/ or exp skull surgery/ or exp stomach surgery/ or exp second look surgery/ or exp male genital system surgery/ or exp vitreoretinal surgery/ or exp abdominal surgery/ or exp colorectal surgery/ or exp open heart surgery/ or exp spinal surgery equipment/ or exp experimental surgery/ or exp arthroscopic surgery/ or exp oral surgery/ or exp elective surgery/ or exp endoscopic surgery/ or exp "aortic root surgery"/ or exp dental surgery/ or exp heart valve surgery/ or exp coagulation surgery/ or exp artery surgery/ or exp maxillofacial surgery/ or exp orthodontic surgery/ or exp larynx surgery/ or exp laser surgery/ or exp "head and neck surgery"/ or exp plastic surgery/ or exp retinal detachment surgery/ or exp new-born surgery/ or exp bariatric surgery/ or exp rectum surgery/ or exp veterinary surgery/ or exp transsphenoidal surgery/ or exp nerve surgery/ or exp pelvis surgery/ or exp eye surgery/ or exp mitral valve surgery/ or exp lung surgery/ or exp surgery/ or exp uterus surgery/ or exp skin surgery/ or exp facial nerve surgery/ or exp endocrine surgery/ or exp hip surgery/ or exp open surgery/ or exp endoscopic endonasal surgery/ or exp bladder surgery/ or exp fetus surgery/ or exp general surgery/ or exp joint surgery/ or exp esophagus surgery/ or exp carotid artery surgery/ or exp gastric bypass surgery/ or exp endoscopic single site surgery/ or exp knee ligament surgery/ or exp ambulatory surgery/ or exp thorax surgery/ or exp retina surgery/ or exp Mohs micrographic surgery/ or exp ureter surgery/ or exp transanal endoscopic surgery/ or exp craniofacial surgery/ or exp orthognathic surgery/ or exp spleen surgery/ or exp coronary artery bypass surgery/ or exp tendon surgery/ or exp middle ear surgery/ or exp ligament surgery/ or exp endodontic surgery/ or exp gynecologic surgery/ or exp vascular surgery/ or exp stereotaxic surgery/ or exp foot surgery/ or exp shoulder surgery/ or exp ultrasound surgery/ or exp endoscopic sinus surgery/ or exp colon surgery/ or exp aesthetic surgery/ or exp brain surgery/ or exp aortic surgery/ or exp minimally invasive surgery/ or exp anus surgery/ or exp ear surgery/ or exp ascending aorta surgery/ or exp failed back surgery syndrome/ or exp prostate surgery/ or exp urinary tract surgery/ or exp emergency surgery/ or exp aortic arch surgery/ or exp descending aortic surgery/ or exp strabismus surgery) |
| Feedback | Ex. Feedback OR Feedback Systems |
| search limits | |
| Search limited to | Keyword and Mesh word search |
| Language | English |
| Year of publication | No restriction |

**Appendix 2: Classification of implementation strategies, ERIC framework (adapted from Waltz and colleagues^17^)**

Implementation strategies are ‘methods and techniques used to enhance the adoption, implementation and sustainability of a clinical programme, practice or intervention’^18^

| Implementation strategy domain | Discrete strategies within the domains |
| --- | --- |
| 1. Use Evaluative and Iterative Strategies (n=10 available strategies) | Assess for readiness and identify barriers and facilitators (1), Audit and provide feedback (2), Conduct cyclical small tests of change (3), Conduct local needs assessment (4), Develop a formal implementation blueprint (5), Develop and implement tools for quality monitoring (6), Develop and organize quality monitoring systems (7), Purposely re-examine the implementation (8), Stage implementation scale up (9), Obtain and use patients/consumers and family feedback (10) |
| 1. Provide Interactive Assistance   (n=4 available strategies) | Provide local technical assistance (11), Facilitation (12), provide clinical supervision (13), Centralize technical assistance (14) |
| 1. Adapt and Tailor to Context (n=4 available strategies) | Use data experts (15), use data warehousing techniques (16), Promote adaptability (17), Tailor strategies (18) |
| 1. Develop Stakeholder Interrelationships (n=17 available strategies) | Develop an implementation glossary (19), Model and simulate change (20), Capture and share local knowledge (21), Conduct local consensus discussions (22), Build a coalition (23), Develop academic partnerships (24), Identify early adopters (25), Inform local opinion leaders (26), Involve executive boards (27), Obtain formal commitments (28), Promote network weaving (29), Use advisory boards and workgroups (30), Use an implementation advisor (31), Visit other sites (32), Identify and prepare champions (33), Recruit, designate and train for leadership (34), Organize clinician implementation team meetings (35) |
| 1. Train and Educate Stakeholders (n=11 available strategies) | Provide ongoing consultation (36), make training dynamic (37), conduct educational meetings (38), conduct educational outreach visits (39), conduct ongoing training (40), create a learning collaborative (41), develop educational materials (42), distribute educational materials (43), Shadow other experts (44), Work with educational institutions (45), Use train-the-trainer strategies (46) |
| 1. Support Clinicians (n=5 available strategies) | Develop resource sharing agreements (47), remind clinicians (48), revise professional roles (49), facilitate relay of clinical data to providers (50), Create new clinical teams (51) |
| 1. Engage Patients and Service Users (n=5 available strategies) | Increase demand (52), intervene with patients/consumers to enhance uptake and adherence (53), involve patients/consumers and family members (54), prepare patients/consumers to be active participants (55), Use mass media (56) |
| 1. Utilize Financial Strategies (n=9 available strategies) | Access new funding (57), alter incentive/allowance structures (58), alter patient/consumer fees (59), develop disincentives (60), Fund and contract for the clinical innovation (61), make billing easier (62), Place innovation on fee for service lists/formularies (63), use capitated payments (64), Use other payment schemes (65) |
| 1. Change Infrastructure (n=8 available strategies) | Change accreditation or membership requirements (66), Mandate change (67), start a dissemination organization (68), change service sites (69), change liability laws (70), change physical structure and equipment (71), change record systems (72), Create or change credentialing and/or licensure standards (73). |

*Note: the number in brackets after each listed implementation strategy corresponds to the numbers listed on the horizontal axis of Figure 2, where the listed strategies are mapped across all studies of the review.*

**Appendix 3: Implementation outcomes framework ^18^**

| **Implementation outcome** | **Definition** |
| --- | --- |
| 1. **Acceptability** | Perception amongst stakeholder’s new intervention is agreeable |
| 1. **Adoption** | Intention to apply or application of new intervention |
| 1. **Appropriateness** | Perceived relevance of intervention to a setting, audience, or problem |
| 1. **Feasibility** | Extent to which an intervention can be applied |
| 1. **Fidelity** | Extent to which an intervention gets applied as originally designed / intended |
| 1. **Implementation costs** | Costs of the delivery strategy, including the costs of the intervention itself |
| 1. **Coverage** | Extend to which eligible patients/population actually receive intervention |
| 1. **Sustainability** | Extent to which a new intervention becomes routinely available / is maintained post-introduction |
