# Supplementary material for: Use of Feedback Data to Reduce Surgical Site Infections and Optimize Antibiotic Use in Surgery: A Systematic Scoping Review
Source: Ann Surg. 2021 Apr 20;275(2):e345–52. doi: 10.1097/SLA.0000000000004909 (PMC8746888; doi:10.1097/SLA.0000000000004909)
Supplement: Supplemental Digital Content [file ansu-275-e345-s002.docx]

**Supplementary file -1**

**List of tables & boxes**

Table A: Characteristics of the included studies

| **S. No** | **Author, Year, Country** | **Surgical Specialty** | **Study Design** | **Study Setting** | **Study duration** | **Intervention description** | **Primary Outcome** | **Secondary Outcome** | **Study Quality (ICROMS)** |
| --- | --- | --- | --- | --- | --- | --- | --- | --- | --- |
| 1. | Abubakar, 2019 (Nigeria)^35^ | Gynecologic and Obstetric | Non controlled before and after study | Multicenter- two tertiary hospitals | 8 months | Multimodal intervention: protocol development on SAP, educational meetings, audit and feedback | SSI rates (4% to 3.4%) (P=0.722) | SAP compliance increased, (from 14.2% to 43.3%), DDD (16.6 ± 3.6 to 12.8 ± 6.8) | 10 |
| 2**. | Aryio, 2019 (USA)^42^ | Multiple specialties | Systematic review | Studies representing both mono and multi-centers | varied | Summarize implementation strategies to improve adherence with evidence-based SSI prevention interventions, using four-E framework. | Varied clinical and implementation outcomes | varied | NA |
| 3. | Arnold, 2016 (USA)^31^ | General surgery | Non controlled before and after study | Monocenter – 110 beds | 29 months | Audit and feedback intervention, assessment of compliance of guidelines for antibiotic use | Compliance of antibiotic orders (70% - 74%, p=0.02). For adaptive therapy (before and after (90% - 93% p<0.01). | DDD calculated, efficacy of IV vs oral formulations compared, gross pharmaceutical expenditure comparison, proportion of new colonization or infection with MRSA calculated. | 15 |
| 4 | Barwolff, 2016 ^36^(Germany) | Gynecologic and Obstetric | Non controlled before and after study | Multicenter – 26 gynecologic and obstetrics department | 72 months | Surveillance system for HAI, individual SSI rates shared twice in a year (German Nosocomial infection surveillance system) | SSI rates after C- section (2.4% to 1.6%), relative risk 0.63 (95%CI 0.48-0.82) |  | 29 |
| 5 | Bos, 2017 (Netherlands)^22^ | Surgical (general), urologic and Orthopedic wards | Non controlled before and after study | Multicenter (2 centers) | 9 months | Multimodal intervention- educational programme and medication counselling for prescribers. Clinical intervention includes: SAP, antithrombosis, fluid and electrolyte management | Preoperative prophylaxis: 16.3% (pre intervention to 14.1% OR (0.63-1.12) (post intervention) | Nonadherence to 10 guidelines. Usual care period [332/1089 (30.5%)]; intervention period [193/886 (21.8%)] OR 0.64, 95% CI 0.52–0.78 | 22 |
| 6 | Brink, 2017?  (South Africa)^23^ | abdominal surgery, trauma, orthopedic, gynecologic & obstetric, vascular surgery, cardiac surgery, neurosurgery, urology | non- controlled pre and post implementation phase | multicenter study (34 centers) private South African hospitals | 30 months | Multimodal bundle- learning sessions for pharmacists and pharmacy managers on Netcare PAP guidelines, core measures for improvement, formation for multidisciplinary teams to conduct regular QI cycles | SSI rates per 1000 operative procedures 2.46 (95% CI 2.18-2.73) to mean rate of 1.97 (95% CI 1.79-2.15, p< 0.0029) | Compliance: pre intervention 65% post intervention 83% p <0.0001 | 26 |
| 7 | Cassir, 2015 (France)^38^ | neurosurgery | prospective cohort study | Single center | 24 months | Review of medical records by a neurosurgeon and an infection prevention and control team member | SSI rates (5.8% in 2009 to 3% in 2010) p =0.04 |  | 2 |
| 8 | Chang, 2019 (USA)^37^ | cardiac surgery | Mixed methods study | Multicenter – 11 hospitals across 9 states | 3 years | Comprehensive Unit based Safety Programme to improve patient safety culture – 1 Educating staff in the science of safety 2. Identifying safety hazards, 3. Assigning senior executives to partner with local QI teams 4. Learning from one defect of system hazard a month and 5. Implementing teamwork tools | SSI rate: (Year 1- Year 3) (2.45, p= .535 to 2.05, p=0.188), CLABSI rate (1.5, p=0.690 to 1.23, p= 0.609) | Patient safety culture: Improvement from Time2 to Time 3 (proportion odd ratio: 1.69 to 2.01).  Staff less likely to respond to feedback and communication about error domain (POR: 0.56 to 0.54). | 26 |
| 9 | Dyck, 2019 (Canada)^24^ | Trauma and orthopedic surgery | non- controlled pre and post implementation phase | multicenter – 3 acute care community hospitals | 60 months | Review of records by IPC staff following one year of surveillance for patients undergoing elective knee or hip arthroplasty | SSI rates (combined hip and knee) 1.48-0.93 p<.005 |  | 17 |
| 10 | Gastmeier, 2005 (Germany)^25^ | Trauma and orthopedic surgery | Retrospective study comparing surveillance | multicenter – 14 centers | 36 months | National surveillance of SSI | SSI reduction with hip procedures with a OR = 0.57; CI 95% (0.42- 0.78). Not significant for knee prosthesis (0.73 955 CI 0.43-1.26) |  | 11 |
| 11. | Geubbels, 2006 (Netherlands)^26^ | Abdominal, trauma and orthopedic surgery, gynecologic and obstetric, vascular surgery | Non controlled before and after study | multicenter SSI surveillance – 37 centers | 60 months | Multimodal intervention– guideline development for hospital surveillance, organization, collection and dissemination of surveillance data on SSI rates, workshops to discuss obstacles in surveillance system (PREZIES- Dutch surveillance system) | SSI rate remained stable for first three years and dropped to 3.3% in 4^th^ year and 1.8% in last surveillance year | Risk of infection reduced for patients undergoing surgery in 4^th^ surveillance year (RR 0.69, 95%CI = 0.52-0.89) and decreased further in 5^th^ year (RR 0.43) CI= 0.24-0.76 | 26 |
| 12. | Jeannette, 2015 (Canada)^39^ | Pediatric surgery | Non controlled before and after study | Single center | 2 years + and follow up 2 months | Multimodal intervention – task force development to review SAP guidelines, computerized verification alerts added, surgeons received feedback | Antibiotic indication improved from 81% (345/426) to 94% (188/200; P < 0.001). | Surgical procedures with prophylaxis indicated, complete  compliance with the guideline improved from 26.2% (49/187) to  53.2% (66/200; P< 0.001). | 16 |
| 13. | Nessim, 2012 (Canada)^32^ | colorectal and hepatic-pancreatic-biliary (HPB) surgical oncology | Qualitative study | Single center – large urban tertiary hospital | 5 months | Quality improvement initiatives including WHO surgical safety checklist implementation, measures to implement and monitor SI prevention – normothermia, razor replaced by clippers in OR, electronic charting system and installation of OR computers for reliable data collection | Individualized feedback does not promote multi-disciplinary work and affects sustainability. | Increase in compliance with all 3 best practices (bundled) from 40% to 76%), as well as a decrease in colorectal SSI rates from 32.3% to 13.4% and HPB SSI rates from 20.0% to 2.9%. | 19 |
| 14. | Rodriuguez, 2006 (Spain)^33^ | all patients undergoing abdominal hysterectomy | Non controlled before and after study (prospective surveillance) | Single center | 5 years | Skin preparation, SAP, hair removal. | The WI rate improved from 10.7% (95% CI: 5.8-15.6) in 1999 to 6% (243.9%) in 2004. P<.001 | The factors associated with WI were albumin (OR, 0.97; 95% CI: 0.94-0.99) and antimicrobial  prophylaxis (OR, 0.08; 95% CI: 0.02-0.32). | 17 |
| 15. | Saied 2015 (Egypt)^27^ | general surgery, orthopedic, gynecologic and obstetric surgery | Non controlled before and after study | Multicenter – 5 tertiary acute care hospitals | pre -intervention survey: Jan and March 2013. Interventions are performed between April and Sept 2013. Post intervention survey between October and Dec 2013 | A six-month AMS intervention aimed to launch appropriate strategies for improving the timing of the first dose before surgery and the duration of antimicrobial therapy for clean and clean contaminated surgeries | drugs for SAP decreased from 843 DOT/1000 patient -days in pre intervention to 335 DOT/1000 patient days in post intervention period in hospital A. Hospital B 1321 to 1090 in Hospital B, 465 to 264 in Hospital C, 669 to 336 in Hospital D. Hospital E had no significant change IRR 0.89; 95% CI, 0.89-1.09; p=.296) |  | 11 |
| 16. | Skyes, 2005 (Australia)^43^ | Any surgical procedure | Retrospective study on SSI rates | Single center – 47,581 surgical procedures | Sept 1988 till October 1990, suspension of program for 15 months. January 1992 until May 1998 | SSI surveillance program implemented (model of SENIC study + post discharge surveillance) | SSI rates, declined over the study period 4.7% (95% CI: 3.9-5.6) in 1988 -1989 to 1.2% (95% CI: 0.8-1.7%) in 2001 (p<.0001) |  | 20 |
| 17. | Van der Slegt, 2013 (Netherlands)^28^ | General surgery, orthopedic, gynecologic and obstetric surgery | Non controlled before and after study | Single center – one large teaching hospital – total 473 surgeries | 3 years | Intervention bundle: 1. Perioperative normothermia 2. Appropriate hair removal 3. Perioperative antibiotic prophylaxis and 4. Discipline in the operating room 5. Feedback | Compliance to bundle improved 10% in 2009 to 60% in 2011 (p< 0.05). SSI rates: 44% lower |  | 20 |
| 18. | Van Kasteren 2005 (Netherlands)^29^ | Orthopedic, vascular, gynecologic and gastro-intestinal surgery | Non controlled before and after study | Multicenter -13 Dutch Hospitals | 2-9 months in each hospital | Multimodal, guidelines on SAP revised, educational meetings conducted, feedback generated, and auditing reports discussed | The number of DDD per 100 population decreased from 121 before to 79 after the intervention. Antibiotic Cost per procedure decreased by 25%. | Duration of prophylaxis – Median time between the first dose at surgical suite and last dose at the ward decreased from 16 hr to 12 h after the intervention. SSI rates across surgical specialties significantly reduced. | 20 |
| 19. | Waters, 2017 (USA)^34^ | Colorectal surgery | Non controlled before and after study | Single center -Tertiary Care hospital (7975 operations data was extracted) | Jan 2008 till Dec 2013 | Each surgeon was provided with biannual feedback on process adherence and surgeon specific feedback on SSI, UTI, DVT, anastomotic leak, 30-day admission, reoperation, and mortality | SSI rates 2008-09 :8+/-1  2010 -11:8+/-1  2012-13: 6+/- 1  p not significant | Reoperations: no substantial decrease in 30 day. Reoperation rate 3.5% to 2.9%  Readmissions: decrease from 7.6 % to 5.6% | 11 |
| 20. | Wilson, 2006 (UK)^30^ | General, orthopedic, cardiac, thoracic, obstetric, Gynecologic, plastic, urologic, vascular, maxillofacial surgery | Retrospective surveillance study | Single center | 4 years | post discharge surveillance and feedback | 9.9% wounds were infected of which 54.7% were detected at discharge. Proportion of patients with superficial and deep infection odds ratio: 0.69 (95% CI 0.57 to 0.83). infection rates fell significantly in orthopedic, cardiac and thoracic) | service cost per patient fell from 1^st^ to 4^th^ year of the study | 11 |
| 21. | Wright, 2016 (Canada)^44^ | Pediatric surgery | Non controlled before and after study | Single center – one children’s hospital | 8 years (unclear) | Multimodal -1. Early monitoring of guideline use (nurses had to chart if the drug was given and the time of administration) 2. Score card was created to monitor the quality and efficiency of activities within perioperative services 3. Use and timing of SAP became part of monthly report 4. Individualized audit and feedback. | Appropriate use of antibiotics in surgery improved over 6 months, repeated audit revealed the compliance with the guidelines for patients to receive and not receive antibiotics was moderately improved. |  | 26 |

Note: The ICROMS assessment was performed on all the studies expect the systematic review^45^

The minimal score requirement for: RCTs = 22, for controlled before and after studies =18, for non-controlled before and after studies =22, qualitative studies =16, surveys =16 and other designs =16.

Table B: Feedback content and delivery mechanisms across included studies

| **Study & Country** | **Format and mode of delivery of feedback** | **Individualized vs. Group feedback** | **Data included in the feedback** | **Frequency of feedback (immediate vs. delayed)** | **Implementation strategies applied (as per the ERIC taxonomy)** | **Feedback recipient/s provider/s** |
| --- | --- | --- | --- | --- | --- | --- |
| 1. Abubakar, 2019 (Nigeria) | oral presentation, poster presentations | Group | Data on SSI incidence rates/ SSI rates is provided | not reported | Audit and provide feedback, develop educational materials, conduct educational meetings | Providers: Pharmacists  Recipients: surgeons |
| 1. Arnold, 2016 (USA) | reports, newsletters | Group and individual | Anti-microbial treatment recommendation for each antimicrobial used, infectious disease related report | Weekly reports and quarterly department specific reports | Audit and provide feedback, provide clinical supervision, remind clinicians | Recipients: internal medicine physicians, general surgeons, chair of pharmacy and therapeutics, infection control and performance improvement departments, chief of staff of medicine and surgery department. |
| 1. Barwolff 2016 (Germany) | Correspondence reference data | Individual | SSI rates | Twice yearly | Audit and provide feedback, Develop and organize quality monitoring systems | Recipients: surgeons and prescribers |
| 1. Boss (2017) Netherlands | Direct and oral feedback | Group | Adherence to pharmacological guidelines, medicines prescribed | Weekly visits | Audit and provide feedback, provide clinical supervision, conduct educational meetings | Providers: Hospital pharmacists  Recipients: surgeons and physicians on surgical wards |
| 1. Brink, 2016 (South Africa) | via email, verbally via learning cycles via journal club, via antimicrobial committee and also through theatre tea rooms | Group | SSI rates (PAP indicators are recorded and fed back) | monthly basis and three-monthly basis | Strategies covering the following domains: Iterative and evaluative strategies; Provide interactive assistance; Adapt and tailor to context; Develop stakeholder interrelationships; Train and educate stakeholders; Support clinicians | Providers: AMS project manager (to pharmacists); pharmacists (to everyone else)  Recipients: Pharmacists (from AMS project managers); multi-disciplinary hospital teams, surgeons, staff nurses and anesthesiologists via journal club. |
| 1. Cassir 2015 (France) | Not reported | Group (implied) | SSI incidence | quarterly | Audit and provide feedback | Recipients: surgical team |
| 1. Chang, 2019 (USA) | Meetings and coaching sessions | Group (implied) | Defects in the CUSP (Comprehensive Unit based Safety programme) | unclear | Strategies covering domains: Iterative and evaluative strategies; Provide interactive assistance; Adapt and tailor to context; Develop stakeholder interrelationships; Train and educate stakeholders | Not clear |
| 1. Dyck 2019 (Canada) | Reports and infographics | Group | Infection rates, pre-operative antibiotics | Quarterly and semi-regularly | Audit and provide feedback, facilitate relay of clinical data to providers | Recipients: Regional orthopedic standards and quality committee, hospital site nursing and surgical leadership, IPC doctors, surgeons, nurses and other and front-line staff |
| 1. Gastmeier 2005 (Germany) | Not reported | Not reported | SSI rates | Twice yearly | Audit and provide feedback; Conduct ongoing training; mandate change | Not reported |
| 1. Geubbels 2006 (Netherlands) | Not reported | Not reported | SSI rates | Not reported | Audit and provide feedback; Conduct ongoing training; mandate change; Recruit, designate, and train for leadership; Capture and share local knowledge | Providers: IPC physicians  Recipients: surgeons, operating room staff, surgical staff, managers, IPC committee |
| 1. Jeannette, 2015 Canada | Email notification by surgical information system | Individual and group | Antibiotic with/without indication, antibiotics given 60 mins prior to incision | Next day and monthly basis | Strategies covering domains: Iterative and evaluative strategies; Develop stakeholder interrelationships; Train and educate stakeholders; support clinicians, change infrastructure | Recipients: division’s additional departments, surgeons |
| 1. Nessim, 2012 Canada | Case specific audit and feedback sheet given | Individualized to clinicians and team-specific feedback sheets to other recipients (group level) | Case-specific compliance with each best practice for procedures from the previous month | Monthly basis | Strategies covering domains: Iterative and evaluative strategies; Train and educate stakeholders; change infrastructure | Recipients: surgeons, anesthesiologists, nursing groups and residents |
| 1. Rodriguez, 2006 (Spain) | Not reported | Not reported | Wound infection rates | Annually | Audit and provide feedback | Recipients: surgeons |
| 1. Saied 2015 (Egypt) | Oral feedback | Group and individual (implied) | Information on suboptimal timing of the first dose, noted suboptimal timing of surgical prophylaxis, information on the antibiotics prescribed | Not clear | Strategies covering the following domains: Iterative and evaluative strategies; Provide interactive assistance; Develop stakeholder interrelationships; Train and educate stakeholders; Support clinicians | Provider: a senior surgeon (‘champion’)  Recipients: all prescribers responsible for surgical patients |
| 1. Skyes 2005 (Australia) | Reports which included tabular and graphical data along with a patient’s operative profile of all infected patients for the specified surgical unit | Not clear | Summary of infection rates | 6 monthly reports | Audit and provide feedback; change record systems | Recipients: surgeons, operating room and surgical ward staff |
| 1. Van der Slegt, 2013 Netherlands | Newsletter | Individualized feedback when bundle adherence at risk and also a group level feedback | information on results of the bundle compliance and recommendations for improvement | 3 monthly newsletter | Multi modal intervention including feedback; Organize clinician implementation team meetings | Recipients: multi-disciplinary perioperative team consisting of surgeons, anesthesiologists, head of operating room, OR personnel and infection prevention control personnel.  Personal feedback provided to surgeons and clinicians |
| 1. Van Kasteren 2005 Netherlands | Meetings where recommendations for local improvement were discussed | Group | Quality of prophylaxis | Not reported | Audit and provide feedback, conduct educational meetings | Recipients: surgeons, anesthesiologists, pharmacists, microbiologists, nurses and local antibiotic committee |
| 1. Waters, 2017 USA | Wherever possible, the data is presented in a year-to-date and multiple-year cumulative format | Individual and also aggregated at departmental level (group level) | Individualized rates, departmental level rates, rates comparing historical departmental level estimates, adherence to process measures and data in adverse events | Biannually | Audit and provide feedback | Recipients: colon and rectal surgeons (8 of them were given feedback) |
| 1. Wilson, 2006 UK | Not reported | Individually (data of others anonymized) and at group level | Wound infection rates | 3- 6 monthly individually to surgeons and to infection prevention committee twice yearly | Strategies covering the following domains: Iterative and evaluative strategies; Adapt and tailor to context; Develop stakeholder interrelationships; change infrastructure | Recipients: surgeon, the hospital administration, infection prevention and control committee surgical wards |
| 1. Wright 2016 Canada | Via automated emails | Individual | Data on antibiotic indication and subsequent appropriateness. Additionally, it included incorrect matching of surgical procedure with guideline, incorrect charting by nurses and incorrect indication of guidelines to receive or not receive antibiotics | Following each operation | Strategies covering the following domains: Iterative and evaluative strategies; Provide interactive assistance; Adapt and tailor to context; Develop stakeholder interrelationships; Train and educate stakeholders; Support clinicians; change infrastructure | Recipients: surgeons and anesthesiologists |

This systematic review^45^ study is not included in this table as feedback as a strategy could not be distilled across studies.

Box 1. Abbreviations used across all tables

| AMS | Antimicrobial Stewardship |
| --- | --- |
| ATB use | Antibiotic Use |
| CI | Confidence Interval |
| DDD | Dose per Day for a Drug |
| DVT | Deep Vein Thrombosis |
| ERIC | Expert Recommendations for Implementing Change |
| HAI | Hospital Acquired Infections |
| SSI | Surgical Site Infections |
| IPC | Infection Prevention and Control |
| PAP | Perioperative Antibiotic Prophylaxis |
| SAP | Surgical Antibiotic Prophylaxis |
| WHO | World Health Organization |
| UTI | Urinary Tract Infections |
